# Supplementary material for: Astaxanthin Induces Apoptosis in MCF-7 Cells through a p53-Dependent Pathway
Source: Int J Mol Sci. 2024 Jun 28;25(13):7111. doi: 10.3390/ijms25137111 (PMC11241156; doi:10.3390/ijms25137111)
Supplement: Supplementary file 1 [file ijms-25-07111-s001.zip › ijms-3070282-supplementary.pdf]

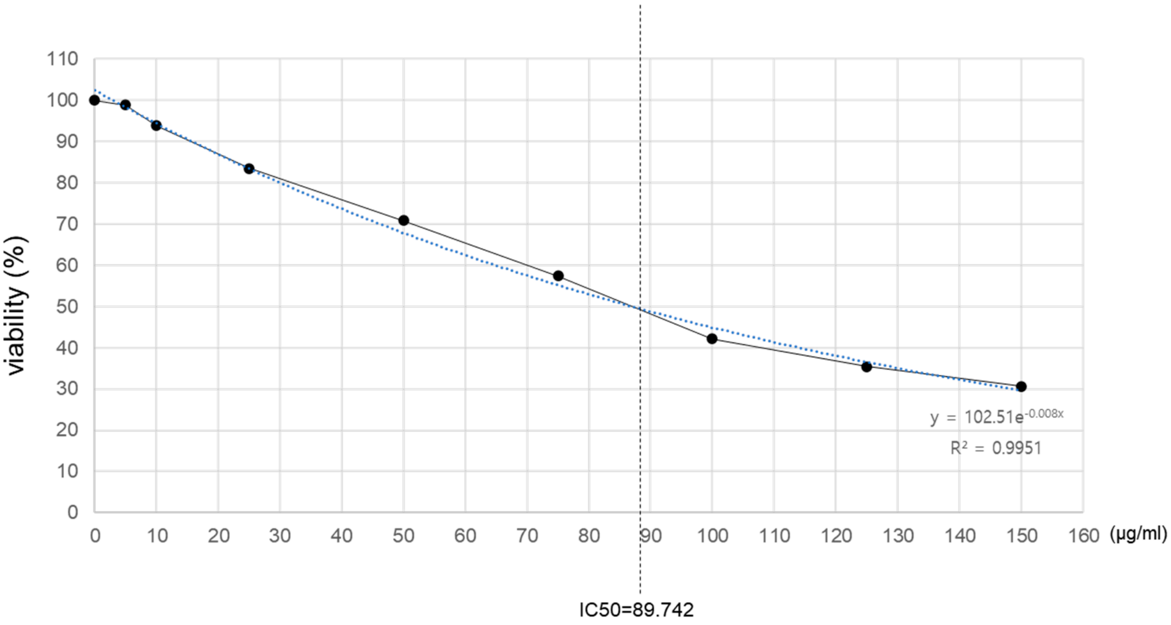

Supplementary Figure S1. The IC50 calculation of AXT.

MCF-7 cells (5×10^3 cells/well) were incubated with the indicated concentration of AXT for 48 h in 96-well plate. After the incubation, PrestoBlue^{TM} was added in the wells, and was measured with spectrophotometer at 540 nm wavelength.
